# The importance of data issues when comparing cystic fibrosis registry outcomes between countries: Are annual review FEV_1_ in the UK only collected when subjects are well?

**DOI:** 10.1111/jep.12967

**Published:** 2018-06-14

**Authors:** Zhe Hui Hoo, Rachael Curley, Michael J. Campbell, Stephen J. Walters, Martin J. Wildman

**Affiliations:** ^1^ School of Health and Related Research (ScHARR) University of Sheffield Sheffield UK; ^2^ Sheffield Adult CF Centre Northern General Hospital Sheffield UK

**Keywords:** clinical epidemiology, cystic fibrosis, respiratory measurement

## Abstract

**Rationale, aims and objective:**

Cross‐country comparisons of cystic fibrosis (CF) outcomes can potentially identify variation in care but are dependent on data quality. An important assumption is that the UK annual review FEV_1_ is only collected during periods of clinical stability. If this assumption does not hold, results of FEV_1_ comparisons may be biased in favour of registries with encounter‐based FEV_1_. We aimed to test the assumption that CF annual reviews in the UK are only performed during periods of clinical stability.

**Method:**

Prospective encounter‐based data collected in Sheffield (*n* = 174) was used to establish whether annual review FEV_1_ were always collected during periods of clinical stability and to determine the group‐level discrepancy between annual review vs best FEV_1_. We then went on to quantify the group‐level discrepancy between annual review and best annual FEV_1_ readings within the UK registry (*n* = 2995) to determine if the differences observed in Sheffield also apply to the wider UK data.

**Results:**

Sheffield results showed a group‐level discrepancy between best and annual review FEV_1_ of −2.5% (95% CI −3.95% to −1.2%) for annual reviews performed during periods of clinical stability (*n* = 50). The group‐level discrepancy is larger at −8.0% (95% CI −11.2% to −4.9%) among annual reviews performed during periods of clinical instability (*n* = 13). Therefore, the magnitude of this group‐level discrepancy is a surrogate for the proportion of clinically stable annual reviews—smaller discrepancy indicates a higher proportion of clinically stable annual reviews and vice versa.

The overall group‐level discrepancy in the UK registry (−5.6%, 95% CI −5.9 to −5.4%) was similar to Sheffield (−6.1%, 95% CI −7.1 to −5.1%). Around 20% of the clinician reviewed, annual reviews in Sheffield were performed during periods of clinically instability.

**Conclusions:**

Annual review FEV_1_ underestimates lung health of adults with CF in the UK and may bias cross‐country comparisons.

## 1 INTRODUCTION

Cystic fibrosis (CF) is an autosomal recessive genetic condition which affects multiple organs, in particular the lungs (resulting in recurrent infections and respiratory failure) and the gastrointestinal tract (resulting in malabsorption of fat and poor growth).^1^ Median predicted survival has improved to over 40 years,^2^ likely because of a combination of factors including better early nutritional supplementation, availability of more efficacious treatment options, and better quality of care. Cross‐country comparisons can contribute to better quality of care. For example, comparisons of nutritional outcomes and survival between the Boston and Toronto CF centres in the 1980s identified the benefits of aggressive nutritional support,^3^ which led to a unified dietary approach for people with CF globally.^4^

Cross‐country CF registry comparison is now an increasingly common method used to identify variation in care and opportunities for system improvement. Examples include the US‐Australia, US‐Canada, and US‐UK comparisons.^5^, ^6^, ^7^ Forced expiratory volume in 1 second (FEV_1_) is an important indicator of lung health among people with CF and has been used an outcome measure in some of the cross‐country comparisons. The recent US‐UK FEV_1_ comparison using 2010 dataset found superior FEV_1_ in the United States, especially among those aged 6 to 25 years.^7^ Higher prescription of inhaled mucolytics among US children was suggested by the investigators as one of the reasons for this difference, although FEV_1_ differences actually persisted across all levels of treatments.^7^, ^8^

Higher FEV_1_ is desirable because it is strongly associated with better survival.^6^ Yet people with CF in the UK were significantly older than the United States,^7^ which suggest that people in the United Kingdom are living longer and have better outcomes. The “pyramid of investigation” provides a systematic approach to understand this apparent paradox and proposes data review as the first step.^9^

In 2010, the US registry collected encounter‐based FEV_1_ whereas the UK registry only collected annual review FEV_1_. The US‐UK comparison used a matching algorithm taking into account seasonality of the UK data to select one FEV_1_ reading from each US study subject.^7^ Only clinically stable FEV_1_ from the United States were matched, because of the assumption that the UK annual review FEV_1_ was always collected “when subjects are well.”^7^ This assumption has never been formally tested.

We investigated this issue by using prospective Sheffield Adult CF Centre encounter‐based FEV_1_ data to establish whether annual review FEV_1_ were always collected during periods of clinical stability. We then went on to repeat our analysis using data from the UK CF registry to determine if the Sheffield findings also apply UK‐wide.

## 2 METHODS AND MATERIALS

Encounter‐based FEV_1_ data were prospectively collected in the Sheffield Adult CF centre between 1 January and 31 December 2016 from every adult who contributed data to the UK CF registry, excluding those who had lung transplantation (*n* = 7) or on ivacaftor (*n* = 13). Annual reviews were performed according to usual practice. In addition, clinicians' opinion of health status and Fuchs' criteria^10^ were recorded during every encounter involving clinician review, including outpatient clinics, ward reviews, and home visits. FEV_1_ readings were deemed to be taken in a period of clinical stability if there was no exacerbation, no requirement for intravenous antibiotics, and ≤3 Fuchs' symptoms present. Every annual review FEV_1_ was matched to another clinically stable FEV_1_ that was closest to the annual review. Mean paired difference and paired *t* test *P*‐value were calculated. Non‐parametric comparisons were also performed to check the robustness of the results.

The UK registry has no “stable FEV_1_” data but collects best FEV_1_ data since 2012 for the European registry.^11^ We therefore quantified the group‐level discrepancy between best FEV_1_ and annual review FEV_1_ in both Sheffield 2016 (best FEV_1_ data in Sheffield represent the highest FEV_1_ reading between 1 January and 31 December 2016) and the UK registry 2014 datasets among people aged ≥16 years to determine if the differences observed in Sheffield also apply UK‐wide.

The UK registry data were collected during annual reviews between 1 January and 31 December 2014. The best FEV_1_ data in the UK registry represent the highest FEV_1_ reading in the 1‐year period prior to the date of annual review (ie if a person had annual review on 1 July 2014, the highest FEV_1_ reading between 1 July 2013 and 1 July 2014 should be that person's “best FEV_1_” for 2014). People who had lung transplantation (*n* = 330) or on ivacaftor (has transformative effect on lung health but unavailable commercially in 2010,^12^ *n* = 281) in the UK registry were excluded. People attending the adult Sheffield CF centre were also excluded to avoid duplicate analysis of the same cohort.

All analyses were performed by using SPSS v22 (IBM Corp, Armonk, NY, USA). Where statistical tests were performed, a *P*‐value <.05 was considered to be statistically significant. Regulatory approval for the analysis of prospective Sheffield data was granted by the National Health Service (NHS) Health Research Authority (IRAS number 210313). National Health Service research ethics approval (Huntingdon Research Ethics Committee 07/Q0104/2) was granted for the UK CF Registry. Under the terms of the NHS ethics approval, the UK CF Trust steering committee approved the use of anonymized data for this analysis.

## 3 RESULTS

A total of 174 adults were included for Sheffield analysis and 2995 adults for the UK CF registry analysis. Adults with and without best FEV_1_ data in the UK CF registry shared similar clinical characteristics (see Table 1).

**Table 1:** Characteristics of adults with cystic fibrosis (CF) for Sheffield in 2016 and other CF centres in the 2014 UK CF registry dataset

Characteristics	2016 Prospective Sheffield Data (*n* = 174)	2014 UK CF Registry Data for Adults With Both Best and Annual Review FEV_1_ (*n* = 2995)^a^	2014 UK CF Registry Data for Adults Without Best FEV_1_ but Annual Review FEV_1_ was Available (*n* = 1320)^a^
Age in years, median, IQR	27 (21‐34)	28 (22‐35)	29 (23‐38)
Female, n, %	84 (48.3)	1336 (44.6)	620 (47.0)
Pancreatic insufficient,^b^ n. %	134 (77.0)	2458 (82.6)^c^	1061 (80.9)^d^
CF related diabetes, n, %	49 (28.2)	979 (32.7)	445 (33.7)
BMI in kg/m^2^, median, IQR	23.4 (20.5‐26.1)	22.2 (20.2‐24.7)	21.9 (19.8‐24.4)
Annual review %FEV_1_,^e^ median, IQR	74.0 (55.0‐88.3)	66.1 (46.3‐84.7)	63.2 (44.2‐84.0)
Best %FEV_1_,^e^ median, IQR	83.0 (63.0‐93.0)	72.1 (52.9‐90.5)	N/A

a Adults receiving care at the Sheffield Adult CF Centre were excluded from this analysis to avoid duplicate analysis of the same cohort. Among 4315 UK CF registry adults with annual review FEV_1_ data in 2014, best FEV_1_ data were available for 2995 adults (69.4%). From 2012 onwards, the UK CF registry collects the best FEV_1_ data because these data are required by the European CF registry.

b Data for pancreatic replacement therapy (PERT) use were obtained. People on PERT were considered “pancreatic insufficient.” People not on PERT were considered “pancreatic sufficient.” PERT use documented as “unknown” is considered as missing data.

c Pancreatic status was missing for 21 (0.7%) of the adults with best FEV_1_ data in the UK CF registry.

d Pancreatic status was missing for 8 (0.6%) of the adults without best FEV_1_ data in the UK CF registry.

e % predicted FEV_1_ was calculated with Knudson equation. For reference, see Knudson RJ, Lebowitz MD, Holberg CJ, Burrows B. Changes in the normal maximal expiratory flow‐volume curve with growth and aging. Am. Rev. Respir. Dis. 1983; 127: 725–34.

There was significant group‐level differences between annual review vs matched clinically stable FEV_1_ in Sheffield (mean −2.9%, 95% CI −3.8% to −1.9%), with similar differences among those with paired readings within 30 days or >30 days apart. Not every episode of clinical instability was accompanied by acute FEV_1_ decline, but variability in FEV_1_ measurements meant that best FEV_1_ would tend to exceed annual review FEV_1_ even when annual review was performed during clinical stability. Sheffield results suggested a group‐level discrepancy between best and annual review FEV_1_ of −2.5% (95% CI −3.9% to −1.2%) for all annual reviews performed during periods of stability (see Table 2). For all annual reviews performed during periods of clinically instability, the group‐level discrepancy was larger at −8.0% (95% CI −11.2% to −4.9%). In Sheffield, whereby 20% of the clinician reviewed annual reviews were performed during periods of clinical instability, the overall group‐level discrepancy between best and annual review FEV_1_ was −6.1% (95% CI −7.5 to −5.1%).

**Table 2:** Summary of parametric FEV_1_ comparisons for the 2016 Sheffield prospectively collected data and the 2014 UK CF registry dataset

Annual Review % FEV_1_ vs Matched Clinically Stable % FEV_1_	Annual Review % FEV_1_ Mean (95% CI)	Matched Clinically Stable % FEV_1_ Mean (95% CI)	Paired Mean Difference in % FEV_1_ (95% CI)	Paired *t* Test *P*‐Value
For the Sheffield cohort in 2016 (*n* = 173)^a^	71.4 (68.1 to 74.7)	74.3 (71.0 to 77.5)	−2.9 (−3.8 to −1.9)	<.001
Paired FEV_1_ readings within 30 days (*n* = 56)	69.5 (63.9 to 75.0)	72.6 (67.0 to 78.2)	−3.2 (−4.3 to −2.0)	<.001
Paired FEV_1_ readings >30 days apart (*n* = 117)	72.4 (68.2 to 76.5)	75.1 (71.1 to 79.1)	−2.7 (−4.0 to −1.4)	<.001
Annual review documented as clinically unstable^b^ (*n* = 13)	68.8 (54.9 to 82.6)	73.1 (58.7 to 87.4)	−4.3 (−8.2 to −0.4)	.033
Status of annual review unknown^c^ (*n* = 110)	69.3 (65.4 to 73.1)	73.5 (69.8 to 77.2)	−4.2 (−5.5 to −3.0)	<.001
Annual review documented as clinically stable^d^ (*n* = 50)	76.8 (69.9 to 83.8)	76.3 (69.2 to 83.5)	0.5 (−0.5 to 1.6)	.329
Annual Review % FEV_1_ vs Best Annual % FEV_1_	Annual Review % FEV_1_ Mean (95% CI)	Best Annual % FEV_1_ Mean (95% CI)	Paired Mean Difference in % FEV_1_ (95% CI)	Paired *t* Test *P*‐Value
For the Sheffield cohort in 2016 (*n* = 174)	71.2 (67.8 to 74.5)	77.2 (74.0 to 80.4)	−6.1 (−7.1 to −5.1)	<.001
Annual review documented as clinically unstable^b^ (*n* = 13)	68.8 (54.9 to 82.6)	76.8 (62.6 to 90.9)	−8.0 (−11.2 to −4.9)	<.001
Status of annual review unknown^c^ (*n* = 111)	68.9 (65.0 to 72.8)	76.3 (72.5 to 80.1)	−7.4 (−8.7 to −6.1)	<.001
Annual review documented as clinically stable^d^ (*n* = 50)	76.8 (69.9 to 83.8)	79.4 (72.7 to 86.0)	−2.5 (−3.9 to −1.2)	<.001
For the UK CF registry dataset in 2014 (*n* = 2995)^e^	66.0 (65.1 to 66.9)	71.7 (70.8 to 72.5)	−5.6 (−5.9 to −5.4)	<.001
16‐17 years^f^ (*n* = 44)	81.2 (73.1 to 89.3)	88.0 (80.3 to 95.7)	−6.8 (−9.4 to −4.3)	<.001
18‐21 years^f^ (*n* = 578)	73.4 (71.4 to 75.4)	80.0 (78.1 to 81.9)	−6.6 (−7.3 to −5.9)	<.001
22‐25 years^f^ (*n* = 582)	68.0 (66.0 to 69.9)	74.4 (72.5 to 76.3)	−6.5 (−7.1 to −5.8)	<.001
26‐29 years^f^ (*n* = 495)	62.7 (60.5 to 64.9)	68.0 (65.8 to 70.2)	−5.3 (−5.8 to −4.7)	<.001
30‐33 years^f^ (*n* = 412)	62.0 (59.7 to 64.4)	66.9 (64.6 to 69.2)	−4.9 (−5.4 to −4.3)	<.001
34‐37 years^f^ (*n* = 287)	62.3 (59.3 to 65.2)	67.5 (64.7 to 70.4)	−5.3 (−6.0 to −4.5)	<.001
38‐41 years^f^ (*n* = 169)	66.1 (62.2 to 70.0)	71.1 (67.3 to 74.8)	−5.0 (−6.0 to −4.0)	<.001
42‐45 years^f^ (*n* = 148)	61.4 (57.6 to 65.3)	66.0 (62.3 to 69.8)	−4.6 (−5.6 to −3.5)	<.001
46‐49 years^f^ (*n* = 111)	64.3 (58.9 to 69.7)	68.9 (63.6 to 74.3)	−4.6 (−6.3 to −3.0)	<.001
≥50 years^f^ (*n* = 169)	61.4 (57.4 to 65.4)	66.1 (62.2 to 70.0)	−4.7 (−5.5 to −3.9)	<.001

a One person had no clinically stable FEV_1_ in 2016.

b An annual review was deemed “clinically unstable” if clinicians felt exacerbation was present, or if clinicians felt intravenous antibiotics was required, or if ≥4 Fuchs' symptoms were present.

c The health status of an annual review status was “unknown” if the adult with CF was not formally reviewed by a CF clinician during the annual review. Most annual reviews in Sheffield do not involve a formal clinical review.

d An annual review was deemed “clinically stable” if clinicians felt there was no exacerbation, no requirement for intravenous antibiotics, and ≤3 Fuchs' symptoms present.

e Among 4315 UK CF registry adults (adults in Sheffield excluded) with annual review FEV_1_ data in 2014, best annual FEV_1_ data were available for 2995 adults (69.4%).

f These are the same age ranges used in the US‐UK FEV_1_ comparison.^7^

A similar overall group‐level discrepancy of −5.6% (95% CI −5.9% to −5.4%) was observed in the UK registry, suggesting that the proportion of annual reviews performed during periods of clinical instability around the UK was similar to Sheffield. This discrepancy was larger among younger adults, similar to the pattern of FEV_1_ discrepancy observed in the US‐UK comparison.^7^ Similar results were obtained with non‐parametric comparisons (see Table 3), suggesting that our estimates are robust.

**Table 3:** Summary of non‐parametric FEV_1_ comparison for the 2016 Sheffield prospectively collected data and the 2014 UK CF registry dataset

Annual Review % FEV_1_ vs Matched Clinically Stable % FEV_1_	Annual Review % FEV_1_ Median (IQR)	Matched Clinically Stable % FEV_1_ Median (IQR)	Paired Median Difference^a^ in % FEV_1_ (95% CI)	Wilcoxon Signed Rank Test *P* value
For the Sheffield cohort in 2016 (*n* = 173)^b^	74.0 (55.0 to 88.5)	80.0 (58.5 to 89.5)	−3.0 (−4.0 to −2.0)	<.001
Paired FEV_1_ readings within 30 days (*n* = 56)	72.5 (55.8 to 85.0)	78.0 (59.5 to 87.0)	−5.0 (−6.5 to −3.5)	<.001
Paired FEV_1_ readings >30 days apart (*n* = 117)	76.0 (55.0 to 90.0)	80.0 (57.0 to 91.0)	−2.5 (−3.5 to −1.5)	<.001
Annual review documented as clinically unstable^c^ (*n* = 13)	71.0 (49.5 to 91.0)	77.0 (53.5 to 92.0)	−5.0 (−9.0 to 0.0)	.041
Status of annual review unknown^d^ (*n* = 110)	73.5 (53.0 to 85.0)	78.0 (57.8 to 88.0)	−4.0 (−5.5 to −3.0)	<.001
Annual review documented as clinically stable^e^ (*n* = 50)	81.5 (59.0 to 92.3)	82.5 (61.8 to 94.0)	0.5 (−1.0 to 2.5)	.371
Annual Review % FEV_1_ vs Best Annual % FEV_1_	Annual Review % FEV_1_ Median (IQR)	Best Annual % FEV_1_ Median (IQR)	Paired Median Difference^a^ in % FEV_1_ (95% CI)	Wilcoxon Signed Rank Test *P* value
For the Sheffield cohort in 2016 (*n* = 174)	74.0 (55.0 to 88.3)	83.0 (63.0 to 93.0)	−6.5 (−7.5 to −6.0)	<.001
Annual review documented as clinically unstable^c^ (*n* = 13)	71.0 (49.5 to 91.0)	79.0 (58.5 to 100.0)	−8.0 (−11.0 to −4.5)	.002
Status of annual review unknown^d^ (*n* = 111)	73.0 (53.0 to 85.0)	81.0 (62.0 to 91.0)	−7.0 (−8.0 to −6.0)	<.001
Annual review documented as clinically stable^e^ (*n* = 50)	81.5 (59.0 to 92.3)	84.5 (63.0 to 94.3)	−5.5 (−8.0 to −3.5)	<.001
For the UK CF registry dataset in 2014 (*n* = 2995)^f^	66.1 (46.3 to 84.7)	72.1 (52.9 to 90.5)	−6.6 (−6.9 to −6.4)	<.001
16‐17 years^g^ (*n* = 44)	89.3 (59.3 to 102.8)	93.2 (67.6 to 105.8)	−9.2 (−12.1 to −5.8)	<.001
18‐21 years^g^ (*n* = 578)	76.5 (56.8 to 91.5)	82.2 (65.9 to 96.8)	−7.4 (−8.1 to −6.8)	<.001
22‐25 years^g^ (*n* = 582)	68.9 (49.9 to 85.7)	75.7 (58.2 to 91.9)	−7.6 (−8.2 to −7.0)	<.001
26‐29 years^g^ (*n* = 495)	60.4 (43.6 to 80.5)	68.0 (48.9 to 87.0)	−6.3 (−6.9 to −5.8)	<.001
30‐33 years^g^ (*n* = 412)	61.3 (41.3 to 80.7)	67.0 (46.6 to 85.1)	−6.0 (−6.7 to −5.4)	<.001
34‐37 years^g^ (*n* = 287)	60.1 (42.2 to 80.0)	65.0 (49.3 to 83.8)	−6.0 (−6.7 to −5.3)	<.001
38‐41 years^g^ (*n* = 169)	65.7 (45.5 to 85.3)	70.8 (53.6 to 89.5)	−6.0 (−7.2 to −5.0)	<.001
42‐45 years^g^ (*n* = 148)	60.8 (42.4 to 79.6)	65.9 (51.4 to 83.7)	−5.7 (−6.8 to −4.7)	<.001
46‐49 years^g^ (*n* = 111)	62.6 (38.9 to 85.4)	70.2 (47.0 to 90.1)	−5.3 (−7.0 to −4.0)	<.001
≥ 50 years^g^ (*n* = 169)	56.9 (39.5 to 82.2)	61.9 (45.1 to 87.9)	−5.9 (−6.8 to −5.2)	<.001

a The non‐parametric method used to estimate the population paired difference between 2 groups involves first calculating all *n* differences *d*_1_, *d*_2_, ..., *d*_*n*_. We then calculate all possible *n* (*n* + 1)/2 averages of pairs of the differences (*d*_1_ + *d*_2_)/2, (*d*_1_ + *d*_3_)/2 etc. including (*d*_*i*_ + *d*_*i*_)/2 for *i* = 1, 2, …, *n*, and then selecting the median of the averages. This method can also be used to find confidence intervals for this median. For reference, see Campbell MJ, Gardner MJ. Calculating confidence intervals for some non‐parametric analyses. Br Med J 1988; 296: 1454‐6.

b One person had no clinically stable FEV_1_ in 2016.

c An annual review was deemed “clinically unstable” if clinicians felt exacerbation was present, or if clinicians felt intravenous antibiotics was required, or if ≥4 Fuchs' symptoms were present.

d The health status of an annual review status was “unknown” if the adult with CF was not formally reviewed by a CF clinician during the annual review. Most annual reviews in Sheffield do not involve a formal clinical review.

e An annual review was deemed “clinically stable” if clinicians felt there was no exacerbation, no requirement for intravenous antibiotics, and ≤3 Fuchs' symptoms present.

f Among 4315 UK CF registry adults (adults in Sheffield excluded) with annual review FEV_1_ data in 2014, best annual FEV_1_ data were available for 2995 adults (69.4%).

g These are the same age ranges used in the US‐UK FEV_1_ comparison.^7^

## 4 DISCUSSION

This is the first study to empirically demonstrate that annual review FEV_1_ in the United Kingdom were not always collected during periods of clinical stability. We found that the magnitude of group‐level discrepancy between best and annual review FEV_1_ was larger for annual reviews performed during periods of clinical instability, compared with annual reviews performed during periods of stability. Therefore, the magnitude of this group‐level discrepancy is a surrogate for the proportion of clinically stable annual reviews—smaller discrepancy indicates a higher proportion of annual review performed during periods of stability and vice versa. Our results suggest that around 20% of all annual reviews in the United Kingdom may be performed during periods of clinical instability and that annual review FEV_1_ in the UK registry underestimated lung health of adults with CF at a group level by 2% to 4% in comparison to clinically stable FEV_1_.

This may bias the US‐UK FEV_1_ comparison against the UK, because FEV_1_ when stable was the intended comparison metric in that analysis. %FEV_1_ in our analysis was calculated by using Knudson equation but similar results would be obtained with GLI equation because paired difference between 2 FEV_1_ readings was calculated.^13^ Our analysis was restricted among adults due to data availability in Sheffield. Although most of the US‐UK FEV_1_ differences were among younger people, the lack of differences among older adults does not exclude the possibility that lung health at a group level in the United Kingdom was being under‐estimated.

Our analysis cannot conclusively prove that the US‐UK FEV_1_ comparison was biased because some “clinically unstable” FEV_1_ in the United States may be mislabelled as “clinically stable.” However, we speculate that under‐estimation of lung health may be more of a problem with the UK data entry system, which does not have encounter‐based FEV_1_ data. Data are typically only entered once annually in the UK with a mid‐January deadline to complete data entry for preceding year, yet annual reviews are staggered throughout the year due to capacity issues. Around 40% of annual reviews are performed during the final quarter of the year,^7^ when exacerbation risks are higher.^14^ If people were unwell when they turn up for annual reviews in the final quarter of the year, the choice would be between completing the annual review anyway or risk missing out on data entirely. Data are entered throughout the year in the United States with no risk of missing data when people turn up unwell for a particular clinical encounter. A previous audit in 2012 also found that data included in the US registry were highly accurate.^15^ Indeed, the distribution of stable FEV_1_ data in the US registry (spread evenly throughout the calendar year) is clearly different from the distribution of annual review FEV_1_ data in the UK registry (clear seasonality with higher proportion of data entered in the final quarter of the year),^7^ suggesting inherent differences between these 2 metrics. In addition, our analysis demonstrated that the magnitude of group‐level discrepancy between best and annual review FEV_1_ was larger among younger compared with older adults, which suggests that the bias from annual review FEV_1_ was greater among younger adults. This correlates with the FEV_1_ differences by age as observed in the US‐UK FEV_1_ comparison.

Of note, results of other cross‐country comparisons also provide circumstantial evidence that annual FEV_1_ data may be under‐estimating lung health of people with CF in comparison to encounter‐based FEV_1_ data. The 2003 US‐Australia comparison found greater height and weight percentiles among Australian children (suggesting better nutritional outcomes), which is not surprising given that Australian children were much more likely to be diagnosed after newborn screening (65.8%) compared with US children (7.2%).^5^ Australia also delivered more aggressive treatment for pulmonary exacerbations,^5^ which contributes to better lung health.^16^, ^17^, ^18^ Despite the very strong correlation between nutritional outcomes and lung health,^19^, ^20^, ^21^ FEV_1_ were actually similar between Australian and US children.^5^ In fact, Australian children had significantly lower FEV_1_ after adjusting for the mode of diagnosis.^5^ In 2003, the US registry started collecting encounter‐based FEV_1_ data whilst the Australian registry was collecting FEV_1_ data annually.^5^ It may be that annual FEV_1_ in Australia was under‐estimating the lung health of Australian children, which could explain the disconnect between nutritional outcomes and lung health observed in the US‐Australia comparison.

Differences in outcomes detected by registry comparisons attract significant attention; hence, a rigorous process should be adopted to interpret the results. The “pyramid of investigation” model advocates an incremental approach to understand outcome variation, starting with data review and only inferring differences in the quality of care (eg mucolytic prescriptions) where data are robust. Attention should be paid to differences in data collection systems because systematic bias in data cannot be easily controlled with statistical methods, even for objective outcomes, e.g. survival.^22^ Best FEV_1_ may be more reliable than annual review FEV_1_ but may still under‐estimate lung health if these data were only collected once a year, as suggested by the US‐Australia comparison. Indeed, best FEV_1_ data are most robust if all FEV_1_ readings are recorded in a single database, such that the highest reading over a given time period can be automatically and accurately identified. Harmonization of data collection system for CF registries around the world using encounter‐based data entry would enable more accurate cross‐country comparisons and also allow the use of other potentially more sensitive metrics such as FEV_1_ variability for comparison.^23^

Systematic data differences should be considered when analysing data and interpreting results from cross‐country registry comparisons. We have demonstrated that UK annual reviews are not always collected during periods of clinical stability. This has potential impact on comparisons with the US registry that collects encounter‐based FEV_1_.

## CONFLICT OF INTEREST

M. J. W. is the Chair of the UK CF Registry Research Committee and has argued in favour of shifting the UK CF registry to an encounter‐based data entry system. Other co‐authors have no conflicts of interest to declare.

## FUNDING

This report is independent research arising from a Doctoral Research Fellowship, Zhe Hui Hoo, DRF‐2014‐07‐092) supported by the National Institute for Health Research. The views expressed in this publication are those of the author (s) and not necessarily those of the NHS, the National Institute for Health Research, or the Department of Health.

## References

[1] Elborn JS . Cystic fibrosis. Lancet. 2016;388(10059):2519‐2531. 27140670 10.1016/S0140-6736(16)00576-6

[2] MacKenzie T , Gifford AH , Sabadosa KA , et al. Longevity of patients with cystic fibrosis in 2000 to 2010 and beyond: survival analysis of the Cystic Fibrosis Foundation patient registry. Ann Intern Med. 2014;161(4):233‐241. 25133359 10.7326/M13-0636 PMC4687404

[3] Corey M , McLaughlin FJ , Williams M , Levison H . A comparison of survival, growth, and pulmonary function in patients with cystic fibrosis in Boston and Toronto. J Clin Epidemiol. 1988;41(6):583‐591. 3260274 10.1016/0895-4356(88)90063-7

[4] Stallings VA , Stark LJ , Robinson KA , et al. Evidence‐based practice recommendations for nutrition‐related management of children and adults with cystic fibrosis and pancreatic insufficiency: results of a systematic review. J Am Diet Assoc. 2008;108(5):832‐839. 18442507 10.1016/j.jada.2008.02.020

[5] Martin B , Schechter MS , Jaffe A , Cooper P , Bell SC , Ranganathan S . Comparison of the US and Australian cystic fibrosis registries: the impact of newborn screening. Pediatrics. 2012;129(2):e348‐e355. 22250024 10.1542/peds.2011-0567

[6] Stephenson AL , Sykes J , Stanojevic S , et al. Survival comparison of patients with cystic fibrosis in Canada and the United States: a population‐based cohort study. Ann Intern Med. 2017;166(8):537‐546. 28288488 10.7326/M16-0858 PMC5467971

[7] Goss CH , MacNeill SJ , Quinton HB , et al. Children and young adults with CF in the USA have better lung function compared with the UK. Thorax. 2015;70(3):229‐236. 25256255 10.1136/thoraxjnl-2014-205718 PMC4838510

[8] Goss CH . Country to country variation: what can be learnt from national cystic fibrosis registries. Curr Opin Pulm Med. 2015;21(6):585‐590. 26390334 10.1097/MCP.0000000000000208

[9] Mohammed MA , Rathbone A , Myers P , et al. An investigation into general practitioners associated with high patient mortality flagged up through the Shipman inquiry: retrospective analysis of routine data. BMJ. 2004;328(7454):1474‐1477. 15205291 10.1136/bmj.328.7454.1474 PMC428518

[10] Fuchs HJ , Borowitz DS , Christiansen DH , et al. Effect of aerosolized recombinant human DNase on exacerbations of respiratory symptoms and on pulmonary function in patients with cystic fibrosis. The Pulmozyme Study Group N Engl J Med. 1994;331(10):637‐642. 10.1056/NEJM199409083311003 7503821

[11] Viviani L , Zolin A , Mehta A , Olesen HV . The European Cystic Fibrosis Society Patient Registry: valuable lessons learned on how to sustain a disease registry. Orphanet J Rare Dis. 2014;9:81. 24908055 10.1186/1750-1172-9-81 PMC4066270

[12] Ramsey BW , Davies J , McElvaney NG , et al. A CFTR potentiator in patients with cystic fibrosis and the G551D mutation. N Engl J Med. 2011;365(18):1663‐1672. 22047557 10.1056/NEJMoa1105185 PMC3230303

[13] Konstan MW , Wagener JS , VanDevanter DR , et al. Comparison of FEV1 reference equations for evaluating a cystic fibrosis therapeutic intervention. Pediatr Pulmonol. 2017;52(8):1013‐1019. 28672067 10.1002/ppul.23751

[14] Etherington C , Naseer R , Conway SP , Whitaker P , Denton M , Peckham DG . The role of respiratory viruses in adult patients with cystic fibrosis receiving intravenous antibiotics for a pulmonary exacerbation. J Cyst Fibros. 2014;13(1):49‐55. 23891398 10.1016/j.jcf.2013.06.004

[15] Knapp EA , Fink AK , Goss CH , et al. The Cystic Fibrosis Foundation Patient Registry. Design and Methods of a National Observational Disease Registry. Ann Am Thorac Soc. 2016;13(7):1173‐1179. 27078236 10.1513/AnnalsATS.201511-781OC

[16] Johnson C , Butler SM , Konstan MW , Morgan W , Wohl ME . Factors influencing outcomes in cystic fibrosis: a center‐based analysis. Chest. 2003;123(1):20‐27. 12527598 10.1378/chest.123.1.20

[17] Schechter MS , Regelmann WE , Sawicki GS , et al. Antibiotic treatment of signs and symptoms of pulmonary exacerbations: a comparison by care site. Pediatr Pulmonol. 2015;50(5):431‐440. 25530325 10.1002/ppul.23147

[18] Hoo ZH , Campbell MJ , Curley R , Walters SJ , Wildman MJ . Do cystic fibrosis centres with the lowest FEV_1_ still use the least amount of intravenous antibiotics? A registry‐based comparison of intravenous antibiotic use among adult CF centres in the UK. J Cyst Fibros. 2018;17(3):360‐367. 29074367 10.1016/j.jcf.2017.10.005

[19] Zemel BS , Jawad AF , FitzSimmons S , Stallings VA . Longitudinal relationship among growth, nutritional status, and pulmonary function in children with cystic fibrosis: analysis of the Cystic Fibrosis Foundation National CF Patient Registry. J Pediatr. 2000;137(3):374‐380. 10969263 10.1067/mpd.2000.107891

[20] Stephenson AL , Mannik LA , Walsh S , et al. Longitudinal trends in nutritional status and the relation between lung function and BMI in cystic fibrosis: a population‐based cohort study. Am J Clin Nutr. 2013;97(4):872‐877. 23388659 10.3945/ajcn.112.051409

[21] Mauch RM , Kmit AH , Marson FA , Levy CE , Barros‐Filho AA , Ribeiro JD . Association of growth and nutritional parameters with pulmonary function in cystic fibrosis: a literature review. Rev Paul Pediatr. 2016;34(4):503‐509. 27181343 10.1016/j.rppede.2016.02.001 PMC5176073

[22] Sykes J , Stanojevic S , Goss CH , et al. A standardized approach to estimating survival statistics for population‐based cystic fibrosis registry cohorts. J Clin Epidemiol. 2016;70:206‐213. 26434789 10.1016/j.jclinepi.2015.08.026 PMC4889445

[23] Morgan WJ , VanDevanter DR , Pasta DJ , et al. Forced expiratory volume in 1 second variability helps identify patients with cystic fibrosis at risk of greater loss of lung function. J Pediatr. 2016;169:116‐121. 26388208 10.1016/j.jpeds.2015.08.042

